# Predictors of Outcomes in Adolescents With Clinical High Risk for Psychosis, Other Psychiatric Symptoms, and Psychosis: A Longitudinal Protocol Study

**DOI:** 10.3389/fpsyt.2019.00787

**Published:** 2019-12-03

**Authors:** Silvia Molteni, Eleonora Filosi, Maria Martina Mensi, Giulia Spada, Chiara Zandrini, Federica Ferro, Matteo Paoletti, Anna Pichiecchio, Ilaria Bonoldi, Umberto Balottin

**Affiliations:** ^1^Department of Brain and Behavioral Sciences, University of Pavia, Pavia, Italy; ^2^Child Neuropsychiatry Unit, IRCCS Mondino Foundation, Pavia, Italy; ^3^Institute of Psychiatry, Psychology and Neuroscience, King’s College of London, London, United Kingdom; ^4^Neuroradiology Department, IRCCS Mondino Foundation, Pavia, Italy

**Keywords:** attenuated psychosis syndrome, adolescence, transition, functioning, prognosis, ARMS, young people, psychosis

## Abstract

In children and adolescents, schizophrenia is one of the ten main causes of disability-adjusted life years. The identification of people at Clinical High Risk of developing Psychosis (CHR-P) is one of the most promising strategies to improve outcomes. However, in children and adolescents research on the CHR-P state is still in its infancy and the clinical validity of at-risk criteria appears understudied in this population. Furthermore, only few studies have evaluated the psychopathological, neuropsychological, neuroimaging characteristics and, especially, long-term outcomes of adolescents at high risk. We present here the protocol of an innovative longitudinal cohort study of adolescents aged 12-17. The sample will consist of patients admitted to a third level neuropsychiatric unit, belonging to one of the following three subgroups: 1) adolescents with established Diagnostic and Statistical Manual of Mental Disorder–Fifth Edition psychosis, 2) adolescents with CHR-P, and 3) adolescents with psychiatric symptoms other than established psychosis or CHR-P. The primary aim of our study is to evaluate the 2-year prognosis across the three groups. We will measure transition to psychosis (or the stability of the diagnosis of psychosis in the psychotic group), the risk of development of other psychiatric disorders, as well as socio-occupational functioning at outcome. The secondary aim will be to explore the effect of specific predictors (clinical, neuropsychological and neuroimaging factors) on the prognosis. At baseline, 1-year and 2-year follow-up participants will be assessed using standardized semi-structured interviews and instruments. Psychopathological and functioning variables, as well as neuropsychological domains will be compared across the three subgroups. Moreover, at baseline and 2-year follow-up all recruited patients will undergo a 3-Tesla magnetic resonance imaging examination and diffusion tensor imaging parameters will be analyzed. We believe that this study will advance our ability to predict outcomes in underage CHR-P samples. In particular, our data will enable a better understanding of the clinical significance of CHR-P in adolescents, and shed new light on prognostic factors that can be used to refine the prediction of clinical outcomes and the implementation of preventive interventions.

## Introduction

During adolescence, the assessment of psychiatric symptoms and disorders is challenging. During this neurodevelopmental period, youth go through a period of body and psychic transformation and experience profound psychosocial and neurobiological changes (1). Several authors have underlined the difficulty in discriminating between normal behaviors and psychiatric symptoms (2). Normative adolescent experiences (e.g. imaginary audience and personal fable) can make the clinical picture blurred and lead to false positive psychiatric diagnoses, especially if non-validated diagnostic tools are administered and/or the assessment is done by professionals that are not adequately trained (3). In recent years, efforts have been devoted to develop diagnostic instruments and interviews that could help clinicians in differentiating between normal adolescent behaviors and psychiatric symptoms in this age range (4–6).

This is especially important as current research shows that 50% of mental disorders begin prior to 14 years of age and 75% have their onset by the age of 24 (7). Furthermore, retrospective studies highlighted that the vast majority of youth receiving a psychiatric diagnosis had already been diagnosed of at least one mental disorder by the age of 11 (8).

These findings support the need of specifically addressing to this neurodevelopmental period.

In children and adolescents, psychotic disorders are among the ten main causes of disability adjusted life years (9). One of the most promising strategies to improve outcomes for these disorders is to detect symptoms of the emerging disorder in patients at Clinical High Risk for Psychosis (CHR-P hereafter) (10, 11).

Over the last 3 decades, specific psychometric instruments have been developed and validated internationally to detect CHR-P individuals [for a meta-analysis of their prognostic accuracy see (12)]. In adult samples it has been shown that these criteria associated with a 20% 2-year risk of developing psychosis [see eTable 4 in (13)] with the majority of patients who transition going to develop schizophrenia spectrum disorders (14). The level of risk is highest in those meeting the Brief and Limited Intermittent Psychotic symptoms subgroup of the CHR-P criteria (15) and peaks within the first two years (16). CHR-P individuals have an increased probability of developing psychosis that can be related to several environmental risk factors (17, 18). Although there are different psychometric interviews available to identify CHR-P individuals (19), overall they show a comparable prognostic accuracy which is also similar to that of other instruments used in preventive medicine (12).

Beyond the risk of developing psychosis, several other studies have investigated the level of functioning and/or quality of life in CHR-P subjects (20–22) with controversial results. A recent meta-analysis found that CHR-P people have large impairment in functioning and worse quality of life than the healthy control group, similar to those observed in other coded psychiatric disorder (such as bipolar disorder). Moreover, only a small to moderate better functioning and similar quality of life compared with the psychosis group was highlighted (23).

In a recent study (24), the authors identified a factor structure composed of social-cognitive bias, reflective self (self-esteem, resilience, physical anhedonia and social anhedonia), neurocognition and pre-reflective self (magical ideation, perceptual aberration and basic symptoms) factors. These factors were not only different between recent-onset patients with schizophrenia, ultra-high risk for psychosis and healthy controls, but were also associated with baseline quality of life both in CHR-P individuals and psychotic patients.

Overall, the CHR-P field has attracted lot of interest to the point that clinically based operational criteria of attenuated psychosis syndrome (APS) have been introduced in the section III as well as in the main text (page 122) of the Diagnostic and Statistical Manual of Mental Disorder–Fifth Edition (DSM-5) (25–27). The prognostic accuracy of the APS category appears similar to that of CHR-P psychometric instruments, at least in individuals seeking help at specialized early detection clinics (28). Yet, the applicability and prognostic accuracy of the APS in adolescents is mostly undetermined (29, 30). Several studies (31–33) agreed that transition risk to psychosis in adolescents is lower than that in adults, suggesting that the APS could be less specific in youth (34). A recent study has confirmed that age has an effect on conversion rate to psychosis with lower rates in children and adolescents (35). On another hand, children and adolescents APS appear to display a higher range of psychiatric symptoms and disorders and to have a higher risk of future psychiatric hospitalizations as well as lower functioning (36, 37).

Candidate prognostic factors to refine the prediction of clinical outcome may include cognitive and neuropsychological factors (38, 39). Available meta-analyses (40, 41) showed that CHR-P people performed significantly worse in verbal learning, visual learning and speed of processing, which also differentiated between CHR-P subjects that converted to psychosis and the ones that did not transition. However, the prognostic relevance of the factors in underage populations is not known.

For example, in a study conducted in a small sample of CHR-P adolescents the only parameter who differentiate those who converted to psychosis from the ones that do not at 6-years follow up was baseline low IQ (42).

Recently, Lam et al. (43) found that cognitive dimensions are not only important in identifying youth that later convert to psychosis but account also for longitudinal changes in social and occupational functioning.

Other potential prognostic factors may be based on neuroimaging markers (14, 44, 45).

White matter abnormalities have been identified in schizophrenia. It has been hypothesized that the presence of an aberrant cortical network and functional connectivity could play a key etiopathogenetic role in the disorder (46).

To date, only a few studies have been conducted in CHR-P subjects where the integrity of white matter has been analyzed by using diffusion tensor imaging (DTI) technique (47).

In a sample of 68 adolescents (33 CHR-P and 35 healthy controls) a significant reduction of fractional anisotropy of superior cerebellar peduncles was found (48).

Other studies used resting state MRI scans and found alteration in the default mode (49) and salience networks connectivity (50) in CHR-P youth as compared to healthy controls.

The study protocol described here aims at filling these gaps in knowledge, with a longitudinal, broad risk approach, driven by the increasing need to refine the ability to predict different clinical outcomes in this population (51).

## Aims

The primary aim of this study is to evaluate the 2-year prognosis in adolescent patients through three diagnostics groups: 1) with established DSM5 psychosis, 2) with CHR-P, and 3) with other psychiatric disorders other than psychosis or CHR-P. Stability of diagnosis will be evaluated in the patients who already have psychosis at baseline.

Transition to psychosis will be evaluated according to the CAARMS criteria. In more detail, the psychosis threshold will be considered crossed if the score in the Unusual Thought Content, Non-Bizarre Ideas, and Disorganized Speech will be as high as 6 in the global rating scale and the score in the Perceptual Abnormalities will be at least equal to 5 in the global rating scale. Patients will enter the psychosis group only if these symptoms are present for more than 1 week and their frequency is equal or higher than: 3–6 times a week for more than one hour per occasion or daily.

Socio-occupational functioning will be evaluated by means of the Social and Occupational Functioning Assessment Scale (SOFAS) (52).

Development of other psychiatric conditions will be confirmed according to DSM-5 criteria.

The secondary aim is to study the effect of different prognostic factors (clinical factors, including family history, obstetric complications and drug use, neuropsychological and neuroimaging variables) influencing the clinical outcome.

## Methods and Analysis

### Study Design and Population

We propose a longitudinal cohort study. The study will last 5 years in total with a recruitment period of 3 years, and each subject included will be assessed three times in a 2-year time span (baseline, 1-year and 2-year follow-up).

The study will be carried out in a third level center (Mondino Foundation, IRCCS, Pavia, Italy). The Mondino Foundation is a very well known National Specialist third level center that receives referrals in the field of child and adolescent neuropsychiatry from all over Italy (and in particular from the Lombardy region and the district of Pavia).

The sample will consist of adolescent patients aged 12–17 years, consecutively admitted to the inpatient or outpatient psychiatric units. Patients who already had a diagnosis of psychotic disorder (prior to assessment), established cognitive impairment (IQ < 70), neurological disorders, head injuries, or any other medical condition that could justify their psychiatric symptoms will be excluded.

Written informed assent and consent will be asked to both participants and their legal guardians, respectively.

### Procedure

Each adolescent patient admitted to the psychiatric inpatient and outpatient units not presenting any of the exclusion criteria will be asked to take part in the study. The study procedure will be thoroughly explained by a trained psychologist to both patients and their legal guardian, and a written consent will be obtained. Patients will be free to ask additional questions and take their time in order to decide whether to take part or not in the study. Once patients and their caregivers consent to the study, the baseline assessment will take place.

### Baseline and Follow-Up Assessments

#### Baseline

At baseline sociodemographic information and previous medical and psychiatric history (previous psychiatric symptoms or diagnoses, medical/pharmacological or psychotherapy treatment) as well as socio-economic status [Four-Factor Index of Social Status, (53)] will be collected.

Patients will undergo an extensive diagnostic assessment that will include clinical interviews, semi-structured clinical interviews [CAARMS (54, 55); (Structured Clinical Interview for DSM-IV axis I and II, i.e. SCID-I and II (56–58), Kiddie-schedule for Affective Disorder and Schizophrenia, i.e. K-SADS-PL (59, 60)), and self-administered questionnaires administered to both parents and patients (Child Behavior Checklist, i.e. CBCL) (61, 62) and Youth Self Report, i.e. YSR (63)].

Based on this extensive clinical assessment, subjects will be divided into three subgroups: 1) adolescents with psychosis according to CAARMS criteria, 2) youth with other psychiatric symptoms that do not meet CHR-P or psychosis criteria, and 3) youth with other psychiatric symptoms that do not meet CHR-P criteria. The presence of psychiatric comorbidities will be recorded according to the DSM-5.

Self-administered questionnaires focusing on quality of life, distress, and family functioning will be completed by both guardians and parents. The clinician will complete specific scales describing the socio-occupational functioning and severity of the patient.

A thorough neuropsychological examination will be performed focusing on several cognitive domains: IQ, attention, reasoning and problem solving, verbal working memory, non-verbal working memory, verbal learning, and processing speed.

All the tests and questionnaires used are translated and validated into Italian.

A neuroimaging exam will complete the baseline examination. Patients will undergo a 3.0 Tesla magnetic resonance imaging (MRI) scan including a diffusion weighted sequence for DTI analysis (see MRI acquisition and processing section).

#### Follow-Up Assessments

Participants will be reassessed at 1-year and 2-year follow-up. Psychopathological, neuropsychological and functioning measures will be collected in the three subgroups. The same assessment as described in the baseline section will be carried out.

Neuroimaging exam will be performed at 2-year follow-up only.

As this is a naturalistic longitudinal study, the research team will not interfere on the patient’s care and treatment, which will consist of treatments as usual (psychosocial, pharmacological and psychotherapy).

#### Clinical Variables and Instruments

In the present study, the validated Italian version of the (CAARMS) (55) will be used to determine whether enrolled subjects met research criteria for CHR-P.

The CAARMS is a semi-structured interview designed to assess prodromal psychopathology for people at high clinical risk for psychosis. The CAARMS has a total of 27 items, which are clustered in seven subscales, of which the first one is used to identify the CHR-P criteria, as detailed elsewhere (34).

This instrument has been shown to possess good to excellent concurrent, discriminant and predictive validity and excellent inter-rater reliability (54). CAARMS interview will be administered only to patients.

In order to further validate the information obtained by the patient and to assess the presence of comorbidity and other DSM-5 Axis I, Kiddie-Schedule for Affective Disorder and Schizophrenia, i.e. K-SADS-PL (59, 60), interviews will be conducted with both patient and parents separately. Structured Clinical Interview for DSM-IV axis II, i.e. SCID II (57, 58), will be administered to participants in order to verify the presence of personality disorders.

In addition, in order to gain the patient’s and caregivers’ perspectives on emerging problem behaviors, quality of life, perceived distress and family functioning, participants and legal guardians will be asked to fill in the following self-administered questionnaires: Child Behavior Checklist, i.e. CBCL (61) and Youth Self Report, i.e. YSR (63); EuroQoL scale (64, 65); Perceived Stress Scale (66, 67); and Family Adaptability and Cohesion Evaluation Scales (FACES-IV) (68, 69).

All clinical measures will be administered by trained psychologist or neuropsychiatrist and collected both at baseline, 1- and 2-year follow-up.

#### Functioning Variables and Instruments

As one of the aims of this study is to evaluate the long-term prognosis and outcome also in terms of functioning, the level of functioning will be evaluated using the Children’s Global Assessment Scale, i.e. CGAS (70) and the Social and Occupational Functioning Assessment Scale, i.e. SOFAS (52) as well as specific scales for role functioning [Global Functioning: Role scale, i.e. GF:R (71) and social functioning (Global Functioning: Social scale, i.e. GF:S (72, 73)]. We will also use the Clinical Global Impression-Severity (CGI-S) scale (74) to assess overall severity of illness as assessed by clinicians.

These measures will be collected both at baseline, 1-year and 2-year follow-up.

#### Neuropsychological Domains and Instruments/Tests

In this study we aim at evaluating the longitudinal profiles of cognition in adolescents with CHR-P, compared with adolescents with psychosis and youth with other psychiatric symptoms that do not meet CHR-P criteria and to examine the possible role of specific cognitive deficits as predictors of outcome in this population. For this purpose, a trained psychologist will administer at baseline, 1-year follow-up, and 2-year follow-up the following extensive neuropsychological assessment focusing on several cognitive domains.

In particular the following cognitive domains will be explored:

- Intelligence quotient: Wechsler scales (WISC-IV and WAIS-R) (75, 76)
- Reasoning and problem Solving: Elithorn Perceptual Maze Test [BVN 12-18, Batteria di Valutazione Neuropsicologica per l’Adolescenza (77)]
- Abstract reasoning and flexibility (executive function): Wisconsin Card Sorting Test (78)
- Verbal working memory: Letter-Number Sequencing Subtest of the Wechsler Scales (75, 76)
- Non verbal working memory: Corsi Block Task (79)
- Selective auditory and visual attention: BVN 12-18 (77)
- Planning and attention (executive functions, visual learning): Rey–Osterrieth complex figure test (80)
- Verbal learning: Hopkins verbal learning test (81)
- Processing Speed: Coding-Digit Symbol subtest of the Wechsler Scales and Category Fluency of the BVN 12-18 (77)

The whole assessment usually takes approximately 2h.

#### MRI Acquisition and Image Processing

Subjects will be examined on a Siemens Skyra 3 T MR scanner, equipped with a sixteen-channel head coil. The MRI protocol will include a high-resolution 3D T1-weighted sequence (MPRAGE: 160 sagittal slices, with 1mm thickness; TR/TE = 2300/2.98 ms; TI = 900 ms; flip angle = 9°, voxel size 1 mm^3^ isotropic). A high angular resolution diffusion-weighted imaging (HARDI) dataset will be acquired as well, using a single-shot spin-echo echo-planar imaging (SE-EPI) sequence [66 contiguous axial slices acquired in an interleaved order, in-plane resolution = 2.2 mm^2^, slice thickness = 2.2mm, TR/TE = 8300/92 ms, flip angle = 90°, 64 non-collinear diffusion sensitization directions at *b* = 2000 s/mm^2^, 1 at *b* = 0, and an integrated parallel acquisition technique acceleration factor (IPAT) of 2].

Image preprocessing will be performed through the FMRIB Software Library (FSL; http://www.fmrib.ox.ac.uk/fsl/). For each subject, skull stripping will be applied to both the T1-weighted and the diffusion-weighted images (DWIs) using FSL’s brain extraction tool. For the DWI dataset, eddy current distortions and motion artifacts will be corrected by registering each diffusion-sensitized volume to the *b*0 volume with an affine transformation. After tensor diagonalization, whole-brain maps of the four main voxelwise quantitative WM metrics will be obtained [mean diffusivity (MD), fractional anisotropy (FA), axial diffusivity (AD) and radial diffusivity (RD)]. The T1-weighted images will be first registered (rigid body alignment) to the *b*0 volume of the DWI dataset and then to the Montreal Neurological Institute (MNI) standard stereotactic atlas using FSL’s linear and nonlinear registration tool. DTI-derived voxelwise maps will be finally warped to the MNI space by applying the transform estimated for the coregistered T1 image.

Voxel-wise TBSS analysis will be performed using the default parameters in the FSL (82). A mean FA image will be created and thinned to create a mean FA skeleton that represents the centers of all tracts common to both the entire group and the chosen subgroups (see subjects’ section). Each subject’s aligned DTI-derived maps will be then projected onto this skeleton, allowing voxel-wise between-group comparisons. Comparisons will be tested using a two-sample *t*-test adjusting for the subject’s age and sex; correction for multiple comparisons will be applied [family-wise error (FWE), thresholded at p = 0.05.]

Tractography will also be performed to identify the main white matter bundles, including the corticospinal tracts, forceps major and minor, the superior longitudinal fasciculus (SLF), the arcuate fasciculus (AF), the inferior frontal-occipital fasciculus (IFOF), the uncinate fasciculus (UF), the inferior longitudinal fasciculus (ILF). Average FA, AD, and RD will be evaluated along the entire reconstructed tracts.

### Data Analysis Plan

#### Sample Size

Given the results of a preliminary feasibility study done by our group (83), we expect to recruit 60 patients per year. We assume that approximately 20% of them will belong to the psychosis group, while the other 80% will be equally distributed in the other two groups.

As the recruitment period will last 3 years, the total sample will consist of 180 subjects of which 40 suffering from psychosis at baseline. On the basis of our preliminary data we expect a Hazard Ratio of developing psychosis in the CHR-P versus youth with other psychiatric symptoms not meeting CHR-P criteria not lower than 2.

#### Power

Using this Hazard Ratio, a power calculation indicates that a sample size of 180 subjects will be needed to detect a statistically significant difference with over 95% power.

#### Planned Statistical Analysis

Kaplan-Meyer survival analysis will be performed to calculate time-dependent cumulative probability to develop psychosis in the two non-psychotic groups.

Log-rank test will be performed to evaluate statistic significance of the raw risk.

Multivariate Cox regression model would be used to investigate the independent contribution to the probability to develop psychosis of the two diagnostic categories, controlling for all potentially confounding variables. The same model will be adopted to differentiate between confounding variables and variables independently contributing to the prognosis.

To calculate the probability to develop psychosis at 1 year and at 2 year in the different diagnostic groups, Markov chain will be performed.

DTI quantitative WM metrics (MD, FA, AD, and RD) for each patient at baseline and 2-year follow-up will be analyzed through Matlab software. Independent sample *t*-tests will be used to determine if there is a significant longitudinal difference in the three groups.

### Ethics and Dissemination

The study protocol was reviewed and approved by the ethics committee of the Institute and all subjects will provide written informed consent in accordance with the Declaration of Helsinki.

## Discussion

As described above, research on high-risk state, especially APS, is still in its infancy in childhood and adolescents.

The results of our projects will be important in addressing the urgent need for studies in this area as well as criticism against the inclusion of APS diagnosis in DSM-5.

An innovative and important aspect of our study is its longitudinal design. To our knowledge, no previous study has ever evaluated the long-term outcome and clinical course of CHR-P in children and adolescents. Moreover, we have adopted the experimental approach to addresses the concept of a broader risk (84): prognosis encompass not only transition to psychosis, but the development of other DSM-5 diagnoses as well as evaluation of functioning in adolescents at risk.

Characterizing CHR-P subjects and identifying predictors of different clinical and functioning pathways, course and long-term outcomes represent a crucial step to enable risk stratification and personalized, risk-adapted treatment.

In particular, our data will enable a better understanding of the clinical significance of CHR-P and APS diagnosis in this age group. We will also evaluate the stability over time of CHR-P diagnosis and characterize its clinical course and socio-demographic, clinical, neuroimaging, and functioning correlates.

Overall, our data will raise knowledge in this research field by better characterizing clinically and functionally adolescents fulfilling CHR-P criteria. Moreover, it will provide information about CHR-P adolescent patients’ specific needs and, thus, it will allow clinicians and researchers to plan more appropriate treatment options and evidence-based interventions.

## Author Contributions

SM, MP, IB, and UB wrote the manuscript. MM, GS, FF, CZ, AP, and EF revised it critically. SM, EF, AP, MM, and UB contributed to study design and critical evaluation of the protocol. EF and MM are currently involved in data collection. All the authors approved the final manuscript and agreed to be accountable for all the aspects of the study.

## Funding

This research was supported by Italian Ministry of Health (Ricerca Corrente 2017-2019).

## Conflict of Interest

The authors declare that the research was conducted in the absence of any commercial or financial relationships that could be construed as a potential conflict of interest.

## Abbreviations

AD, Axial Diffusivity; AF, Arcuate Fasciculus; APS, Attenuated Psychosis Syndrome; BVN 12-18, Batteria di Valutazione Neuropsicologica per l’Adolescenza (Neuropsychological Evaluation Battery for Adolescence); CAARMS, Comprehensive Assessment of At Risk Mental States; CBCL, Child Behavior Checklist; CGAS, Children’s Global Assessment Scale; GF:R, Global Functioning: Role scale; GF:S, Global Functioning: Social scale; CGI-S, Clinical Global Impression-Severity; CHR-P, Clinical High Risk of developing Psychosis; DSM-5, Diagnostic and Statistical Manual of Mental Disorder–Fifth Edition; DTI, Diffusion Tensor Imaging; DWI, Diffusion-Weighted Images; EuroQoL scale, instrument for measuring quality of life; FA, Fractional Anisotropy; FACES-IV, Family Adaptability and Cohesion Evaluation Scales; FSL, FMRIB Software Library; FWE, Family-Wise Error; HARDI, High Angular Resolution Diffusion-Weighted Imaging; IFOF, inferior frontal-occipital fasciculus; ILF, inferior longitudinal fasciculus; IPAT, Integrated Parallel Acquisition Technique Acceleration Factor; IQ, intelligence quotient; KSADS-P, Kiddie-schedule for Affective Disorder and Schizophrenia; MD, Mean Diffusivity; MNI, Montreal Neurological Institute; MRI, Magnetic Resonance Imaging; RD, Radial Diffusivity; SCID-I and II, Structured Clinical Interview for DSM-IV axis I and II; SE-EPI, Single-Shot Spin-Echo Echo-Planar Imaging; SLF, Superior Longitudinal Fasciculus; SOFAS, Social and Occupational Functioning Assessment Scale; TBSS, Tract-based spatial statistics; UF, Uncinate Fasciculus; WISC-IV, Wechsler Intelligence Scale for Children; WAIS-R, Wechsler Adult Intelligence Scale-Revised; WM, white matter; YSR, Youth Self Report.

## References

[1] Paus T Keshavan M Giedd JN Why do many psychiatric disorders emerge during adolescence? Nat Rev Neurosci (2008) 9:947–57. 10.1038/nrn2513 PMC2762785 19002191

[2] Welsh P Tiffin PA Attitudes of patients and clinicians in relation to the at-risk state for psychosis. Early Interv Psychiatry (2013) 7:361–7. 10.1111/eip.12062 23773477

[3] Carol EE Mittal VA Normative adolescent experiences may confound assessment of positive symptoms in youth at ultra-high risk for psychosis. Schizophr Res (2015) 166:358–9. 10.1016/j.schres.2015.04.043 25986415

[4] Fux L Walger P Schimmelmann BG Schultze-Lutter F The schizophrenia proneness instrument, child and youth version (SPI-CY): practicability and discriminative validity. Schizophr Res (2013) 146:69–78. 10.1016/j.schres.2013.02.014 23473813

[5] Renou S Hergueta T Flament M Mouren-Simeoni M-C Lecrubier Y [Diagnostic structured interviews in child and adolescent’s psychiatry]. L’Encephale (2004) 30:122–34. 10.1016/S0013-7006(04)95422-X 15107714

[6] Matuschek T Jaeger S Stadelmann S Dölling K Grunewald M Weis S Implementing the K-SADS-PL as a standard diagnostic tool: effects on clinical diagnoses. Psychiatry Res (2016) 236:119–24. 10.1016/j.psychres.2015.12.021 26724908

[7] Kessler RC Berglund P Demler O Jin R Merikangas KR Walters EE Lifetime prevalence and age-of-onset distributions of DSM-IV disorders in the national comorbidity survey replication. Arch Gen Psychiatry (2005) 62:593–602. 10.1001/archpsyc.62.6.593 15939837

[8] Jones PB Adult mental health disorders and their age at onset. Br J Psychiatry Suppl (2013) 54:5–10. 10.1192/bjp.bp.112.119164 23288502

[9] Gore FM Bloem PJN Patton GC Ferguson J Joseph V Coffey C Global burden of disease in young people aged 10-24 years: a systematic analysis. Lancet Lond Engl (2011) 377:2093–102. 10.1016/S0140-6736(11)60512-6 21652063

[10] Fusar-Poli P The clinical high-risk state for psychosis (CHR-P), Version II. Schizophr Bull (2017) 43:44–7. 10.1093/schbul/sbw158 PMC5216870 28053129

[11] Cornblatt BA Lencz T Smith CW Correll CU Auther AM Nakayama E The schizophrenia prodrome revisited: a neurodevelopmental perspective. Schizophr Bull (2003) 29:633–51. 10.1093/oxfordjournals.schbul.a007036 14989404

[12] Fusar-Poli P Cappucciati M Rutigliano G Schultze-Lutter F Bonoldi I Borgwardt S At risk or not at risk? A meta-analysis of the prognostic accuracy of psychometric interviews for psychosis prediction. World Psychiatry Off J World Psychiatr Assoc WPA (2015) 14:322–32. 10.1002/wps.20250 PMC4592655 26407788

[13] Fusar-Poli P Cappucciati M Borgwardt S Woods SW Addington J Nelson B Heterogeneity of psychosis risk within individuals at clinical high risk: a meta-analytical stratification. JAMA Psychiatry (2016) 73:113–20. 10.1001/jamapsychiatry.2015.2324 26719911

[14] Fusar-Poli P Borgwardt S Bechdolf A Addington J Riecher-Rössler A Schultze-Lutter F The psychosis high-risk state: a comprehensive state-of-the-art review. JAMA Psychiatry (2013) 70:107–20. 10.1001/jamapsychiatry.2013.269 PMC4356506 23165428

[15] Fusar-Poli P Cappucciati M De Micheli A Rutigliano G Bonoldi I Tognin S Diagnostic and prognostic significance of brief limited intermittent psychotic symptoms (BLIPS) in individuals at ultra high risk. Schizophr Bull (2017) 43:48–56. 10.1093/schbul/sbw151 28053130 PMC5216865

[16] Kempton MJ Bonoldi I Valmaggia L McGuire P Fusar-Poli P Speed of psychosis progression in people at ultra-high clinical risk: a complementary meta-analysis. JAMA Psychiatry (2015) 72:622–3. 10.1001/jamapsychiatry.2015.0094 25901658

[17] Radua J Ramella-Cravaro V Ioannidis JPA Reichenberg A Phiphopthatsanee N Amir T What causes psychosis? An umbrella review of risk and protective factors. World Psychiatry Off J World Psychiatr Assoc WPA (2018) 17:49–66. 10.1002/wps.20490 PMC5775150 29352556

[18] Fusar-Poli P Tantardini M De Simone S Ramella-Cravaro V Oliver D Kingdon J Deconstructing vulnerability for psychosis: meta-analysis of environmental risk factors for psychosis in subjects at ultra high-risk. Eur Psychiatry J Assoc Eur Psychiatr (2017) 40:65–75. 10.1016/j.eurpsy.2016.09.003 27992836

[19] Fusar-Poli P Cappucciati M Rutigliano G Lee TY Beverly Q Bonoldi I Towards a standard psychometric diagnostic interview for subjects at ultra high risk of psychosis: CAARMS versus SIPS. Psychiatry J (2016) 2016:7146341. 10.1155/2016/7146341 27314005 PMC4904115

[20] Addington J Penn D Woods SW Addington D Perkins DO Social functioning in individuals at clinical high risk for psychosis. Schizophr Res (2008) 99:119–24. 10.1016/j.schres.2007.10.001 PMC2292799 18023329

[21] Simon AE Borgwardt S Riecher-Rössler A Velthorst E de Haan L Fusar-Poli P Moving beyond transition outcomes: meta-analysis of remission rates in individuals at high clinical risk for psychosis. Psychiatry Res (2013) 209:266–72. 10.1016/j.psychres.2013.03.004 23871169

[22] Ruhrmann S Paruch J Bechdolf A Pukrop R Wagner M Berning J Reduced subjective quality of life in persons at risk for psychosis. Acta Psychiatr Scand (2008) 117:357–68. 10.1111/j.1600-0447.2008.01152.x 18241303

[23] Fusar-Poli P Rocchetti M Sardella A Avila A Brandizzi M Caverzasi E Disorder, not just state of risk: meta-analysis of functioning and quality of life in people at high risk of psychosis. Br J Psychiatry J Ment Sci (2015) 207:198–206. 10.1192/bjp.bp.114.157115 26329563

[24] Kim HK Park HY Seo E Bang M Song YY Lee SY Factors associated with psychosocial functioning and outcome of individuals with recent-onset schizophrenia and at ultra-high risk for psychosis. Front Psychiatry (2019) 10:459. 10.3389/fpsyt.2019.00459 31293463 PMC6606785

[25] American Psychiatric Association American Psychiatric Association, DSM-5 Task Force. In: Diagnostic and statistical manual of mental disorders: DSM-5. (Washington, DC: American Psychiatric Association) (2013). 10.1176/appi.books.9780890425596

[26] Fusar-Poli P Carpenter WT Woods SW McGlashan TH Attenuated psychosis syndrome: ready for DSM-5.1? Annu Rev Clin Psychol (2014) 10:155–92. 10.1146/annurev-clinpsy-032813-153645 24471375

[27] Carpenter WT Attenuated psychosis syndrome: a new diagnostic class? J Nerv Ment Dis (2015) 203:325–7. 10.1097/NMD.0000000000000283 25919382

[28] Fusar-Poli P De Micheli A Cappucciati M Rutigliano G Davies C Ramella-Cravaro V Diagnostic and prognostic significance of DSM-5 attenuated psychosis syndrome in services for individuals at ultra high risk for psychosis. Schizophr Bull (2018) 44:264–75. 10.1093/schbul/sbx055 PMC5814820 28521060

[29] Fiori Nastro P Schimmelmann BG Gebhardt E Monducci E Resch F Koch E [Challenges in the early detection of psychosis in children and adolescents]. Riv Psichiatr (2012) 47:116–25. 10.1708/1069.11716 22622248

[30] Schimmelmann BG Walger P Schultze-Lutter F The significance of at-risk symptoms for psychosis in children and adolescents. Can J Psychiatry Rev Can Psychiatr (2013) 58:32–40. 10.1177/070674371305800107 23327754

[31] Welsh P Tiffin PA The “at-risk mental state” for psychosis in adolescents: clinical presentation, transition and remission. Child Psychiatry Hum Dev (2014) 45:90–8. 10.1007/s10578-013-0380-z 23584729

[32] Fusar-Poli P Bonoldi I Yung AR Borgwardt S Kempton MJ Valmaggia L Predicting psychosis: meta-analysis of transition outcomes in individuals at high clinical risk. Arch Gen Psychiatry (2012) 69:220–9. 10.1001/archgenpsychiatry.2011.1472 22393215

[33] Ziermans TB Schothorst PF Sprong M van Engeland H Transition and remission in adolescents at ultra-high risk for psychosis. Schizophr Res (2011) 126:58–64. 10.1016/j.schres.2010.10.022 21095104

[34] Cornblatt BA Lencz T Smith CW Olsen R Auther AM Nakayama E Can antidepressants be used to treat the schizophrenia prodrome? Results of a prospective, naturalistic treatment study of adolescents. J Clin Psychiatry (2007) 68:546–57. 10.4088/JCP.v68n0410 17474810

[35] Schimmelmann BG Michel C Martz-Irngartinger A Linder C Schultze-Lutter F Age matters in the prevalence and clinical significance of ultra-high-risk for psychosis symptoms and criteria in the general population: findings from the BEAR and BEARS-kid studies. World Psychiatry Off J World Psychiatr Assoc WPA (2015) 14:189–97. 10.1002/wps.20216 PMC4471976 26043337

[36] Meyer SE Bearden CE Lux SR Gordon JL Johnson JK O’Brien MP The psychosis prodrome in adolescent patients viewed through the lens of DSM-IV. J Child Adolesc Psychopharmacol (2005) 15:434–51. 10.1089/cap.2005.15.434 16092909

[37] Gerstenberg M Hauser M Al-Jadiri A Sheridan EM Kishimoto T Borenstein Y Frequency and correlates of DSM-5 attenuated psychosis syndrome in a sample of adolescent inpatients with nonpsychotic psychiatric disorders. J Clin Psychiatry (2015) 76:e1449–1458. 10.4088/JCP.14m09435 26646040

[38] Palmer BW Savla GN Fellows IE Twamley EW Jeste DV Lacro JP Do people with schizophrenia have differential impairment in episodic memory and/or working memory relative to other cognitive abilities? Schizophr Res (2010) 116:259–65. 10.1016/j.schres.2009.11.002 PMC2818439 19945256

[39] Palmer BW Dawes SE Heaton RK What do we know about neuropsychological aspects of schizophrenia? Neuropsychol Rev (2009) 19:365–84. 10.1007/s11065-009-9109-y PMC2745531 19639412

[40] Hauser M Zhang J-P Sheridan EM Burdick KE Mogil R Kane JM Neuropsychological test performance to enhance identification of subjects at clinical high risk for psychosis and to be most promising for predictive algorithms for conversion to psychosis: a meta-analysis. J Clin Psychiatry (2017) 78:e28–40. 10.4088/JCP.15r10197 28129494

[41] Fusar-Poli P Deste G Smieskova R Barlati S Yung AR Howes O Cognitive functioning in prodromal psychosis: a meta-analysis. Arch Gen Psychiatry (2012) 69:562–71. 10.1001/archgenpsychiatry.2011.1592 22664547

[42] Ziermans T de Wit S Schothorst P Sprong M van Engeland H Kahn R Neurocognitive and clinical predictors of long-term outcome in adolescents at ultra-high risk for psychosis: a 6-year follow-up. PloS One (2014) 9:e93994. 10.1371/journal.pone.0093994 24705808 PMC3976376

[43] Lam M Lee J Rapisarda A See YM Yang Z Lee S-A Longitudinal cognitive changes in young individuals at ultrahigh risk for psychosis. JAMA Psychiatry (2018) 75:929–39. 10.1001/jamapsychiatry.2018.1668 PMC6142925 30046827

[44] Borgwardt S McGuire P Fusar-Poli P Gray matters!–mapping the transition to psychosis. Schizophr Res (2011) 133:63–7. 10.1016/j.schres.2011.08.021 21943556

[45] Fusar-Poli P McGuire P Borgwardt S Mapping prodromal psychosis: a critical review of neuroimaging studies. Eur Psychiatry J Assoc Eur Psychiatr (2012) 27:181–91. 10.1016/j.eurpsy.2011.06.006 21940151

[46] Bora E Fornito A Radua J Walterfang M Seal M Wood SJ Neuroanatomical abnormalities in schizophrenia: a multimodal voxelwise meta-analysis and meta-regression analysis. Schizophr Res (2011) 127:46–57. 10.1016/j.schres.2010.12.020 21300524

[47] Carletti F Woolley JB Bhattacharyya S Perez-Iglesias R Fusar Poli P Valmaggia L Alterations in white matter evident before the onset of psychosis. Schizophr Bull (2012) 38:1170–9. 10.1093/schbul/sbs053 PMC3494044 22472474

[48] Mittal VA Dean DJ Bernard JA Orr JM Pelletier-Baldelli A Carol EE Neurological soft signs predict abnormal cerebellar-thalamic tract development and negative symptoms in adolescents at high risk for psychosis: a longitudinal perspective. Schizophr Bull (2014) 40:1204–15. 10.1093/schbul/sbt199 PMC4193696 24375457

[49] Clark SV Mittal VA Bernard JA Ahmadi A King TZ Turner JA Stronger default mode network connectivity is associated with poorer clinical insight in youth at ultra high-risk for psychotic disorders. Schizophr Res (2018) 193:244–50. 10.1016/j.schres.2017.06.043 PMC5756141 28688741

[50] Pelletier-Baldelli A Bernard JA Mittal VA Intrinsic functional connectivity in salience and default mode networks and aberrant social processes in youth at ultra-high risk for psychosis. PloS One (2015) 10:e0134936. 10.1371/journal.pone.0134936 26252525 PMC4529226

[51] Oliver D Kotlicka-Antczak M Minichino A Spada G McGuire P Fusar-Poli P Meta-analytical prognostic accuracy of the comprehensive assessment of at risk mental states (CAARMS): The need for refined prediction. Eur Psychiatry J Assoc Eur Psychiatr (2018) 49:62–8. 10.1016/j.eurpsy.2017.10.001 29413807

[52] Goldman HH Skodol AE Lave TR Revising axis V for DSM-IV: a review of measures of social functioning. Am J Psychiatry (1992) 149:1148–56. 10.1176/ajp.149.9.1148 1386964

[53] Cirino PT Chin CE Sevcik RA Wolf M Lovett M Morris RD Measuring socioeconomic status: reliability and preliminary validity for different approaches. Assessment (2002) 9:145–55. 10.1177/10791102009002005 12066829

[54] Yung AR Yuen HP McGorry PD Phillips LJ Kelly D Dell’Olio M Mapping the onset of psychosis: the comprehensive assessment of at-risk mental states. Aust N Z J Psychiatry (2005) 39:964–71. 10.1111/j.1440-1614.2005.01714.x 16343296

[55] Fusar-Poli P Hobson R Raduelli M Balottin U Reliability and validity of the comprehensive assessment of the at risk mental state, italian version (CAARMS-I). Curr Pharm Des (2012) 18:386–91. 10.2174/138161212799316118 22239569

[56] First M Spitzer B Robert L Gibbon M Williams J Structured Clinical Interview for DSM-IV Axis I Disorders, Clinician Version (SCID-CV). Washington, D.C: American Psychiatric Press, Inc. (1996). 10.1037/t07827-000

[57] First M Gibbon M Spitzer R Williams J Benjamin L Structured Clinical Interview for DSM-IV Axis II Personality Disorders, (SCID-II). Washington, D.C: Washington, D.C.: American Psychiatric Press, Inc. (1997).

[58] First M Williams J Karg R Spitzer R SCID-5-CV Starter kit, Ed. Cortina Raffaello (2017)

[59] Kaufman J Birmaher B Brent D Rao U Flynn C Moreci P Schedule for affective disorders and schizophrenia for school-age children-present and lifetime version (K-SADS-PL): initial reliability and validity data. J Am Acad Child Adolesc Psychiatry (1997) 36:980–8. 10.1097/00004583-199707000-00021 9204677

[60] Kaufman J Birmaher B Rao U Ryan N K-SADS-PL DSM-5. Intervista diagnostica per la valutazione dei disturbi psicopatologici in bambini e adolescenti, Erickson. (2019)

[61] Achenbach TM Manual for the Child Behavior Checklist/4-18 and 1991 profile. Burlington: Department of Psychiatry, University of Vermont (1991).

[62] D’Orlando F Grassi M Di Blas L Uno studio di validazione del CBCL/6-18 e del TRF/6-18 nella tarda infanzia. G Ital Psicol (2010) 37:919–43. 10.1421/33434

[63] Achenbach TM Manual for the youth self-report and 1991 profile. Burlington: Department of Psychiatry, University of Vermont (1991).

[64] EuroQol Group EuroQol–a new facility for the measurement of health-related quality of life. Health Policy Amst Neth (1990) 16:199–208. 10.1016/0168-8510(90)90421-9 10109801

[65] Balestroni G Bertolotti G [EuroQol-5D (EQ-5D): an instrument for measuring quality of life]. Monaldi Arch Chest Dis Arch Monaldi Mal Torace (2012) 78:155–9. 10.4081/monaldi.2012.121 23614330

[66] Cohen S Kamarck T Mermelstein R A global measure of perceived stress. J Health Soc Behav (1983) 24:385–96. 10.2307/2136404 6668417

[67] Concerto C Conti C Muscatello MR Signorelli MS Zoccali R Coira D Sleep quality, perceived stress, and caffeinated drinks intake in psychiatry residents: a cross-sectional study. J Caffeine Res (2017) 7:18–22. 10.1089/jcr.2016.0014 29404198 PMC5796400

[68] Olson D FACES IV and the circumplex model: validation study. J Marital Fam Ther (2011) 37:64–80. 10.1111/j.1752-0606.2009.00175.x 21198689

[69] Visani E Di Nuovo S Loriedo C Il Faces IV. Il modello circonflesso di Olson nella clinica e nella ricerca, Ed. Angeli Franco (2014).

[70] Shaffer D Gould MS Brasic J Ambrosini P Fisher P Bird H Aluwahlia S A children’s global assessment scale (CGAS). Arch Gen Psychiatry (1983) 40:1228–31. 10.1001/archpsyc.1983.01790100074010 6639293

[71] Niendam T Bearden C Johnson J Cannon T Global Functioning: Role Scale (GF: Role). Los Angeles, CA: University of California (2006).

[72] Auther A Smith C Corblatt B Global Functioning: Social Scale (GF: Social). Glen Oaks, NY: Zucker-Hillside Hospital (2006).

[73] Lo Cascio N Curto M Pasqualetti P Lindau JF Girardi N Saba R Impairment in social functioning differentiates youth meeting ultra-high risk for psychosis criteria from other mental health help-seekers: a validation of the italian version of the global functioning: social and global functioning: role scales. Psychiatry Res (2017) 253:296–302. 10.1016/j.psychres.2017.04.008 28412612

[74] Guy W Clinical global impression (C.G.I.). Ecdeu Assessment Manual for Psychopharmacology. US Dept Health, Education, and Welfare publication (ADM) 76-338. Rockville MD: National Institute of Mental Health (1976) 218-22.

[75] Wechsler D (1997). WAIS-R Wechsler Adult Intelligence Scale-Revised, Ed. Giunti O. S. 10.1037/t49755-000

[76] Wechsler D WISC-IV Wechsler Intelligence Scale for Children IV Nuovo modello teorico, nuovi subtest, nuovi punteggi, nuove norme: il perfezionamento dell’eccellenza. (2012).

[77] Gugliotta M Bisiacchi P Cendron M Tressoldi P Vio C Batteria di Valutazione Neuropsicologica per l’Adolescenza, Trento: Erickson (2009).

[78] Nelson HE A modified card sorting test sensitive to frontal lobe defects. Cortex J Devoted Study Nerv Syst Behav (1976) 12:313–24. 10.1016/S0010-9452(76)80035-4 1009768

[79] Orsini A Schiappa O Grossi D Sex and cultural differences in children’s spatial and verbal memory span. Percept Mot Skills (1981) 53:39–42. 10.2466/pms.1981.53.1.39 7290882

[80] Caffarra P Vezzadini G Dieci F Zonato F Venneri A Rey-Osterrieth complex figure: normative values in an Italian population sample. Neurol Sci Off J Ital Neurol Soc Ital Soc Clin Neurophysiol (2002) 22:443–7. 10.1007/s100720200003 11976975

[81] Woods SP Scott JC Conover E Marcotte TD Heaton RK Grant I Test-retest reliability of component process variables within the Hopkins Verbal Learning Test-Revised. Assessment (2005) 12:96–100. 10.1177/1073191104270342 15695747

[82] Smith SM Jenkinson M Johansen-Berg H Rueckert D Nichols TE Mackay CE Tract-based spatial statistics: voxelwise analysis of multi-subject diffusion data. NeuroImage (2006) 31:1487–505. 10.1016/j.neuroimage.2006.02.024 16624579

[83] Spada G Molteni S Pistone C Chiappedi M McGuire P Fusar-Poli P Identifying children and adolescents at ultra high risk of psychosis in Italian neuropsychiatry services: a feasibility study. Eur Child Adolesc Psychiatry (2016) 25:91–106. 10.1007/s00787-015-0710-8 25925786

[84] Hartmann JA Nelson B Spooner R Paul Amminger G Chanen A Davey CG Broad clinical high-risk mental state (CHARMS): methodology of a cohort study validating criteria for pluripotent risk. Early Interv Psychiatry (2017). 13(3):379–86. 10.1111/eip.12483 28984077

